# Assessment of Liver Fibrosis: Noninvasive Means

**DOI:** 10.4103/1319-3767.43273

**Published:** 2008-10

**Authors:** Thierry Poynard, Rachel Morra, Patrick Ingiliz, Françoise Imbert-Bismut, Dominique Thabut, Djamila Messous, Mona Munteanu, Julien Massard, Yves Benhamou, Vlad Ratziu

**Affiliations:** Service d'Hépato-Gastroentérologie, Groupe Hospitalier Pitié-Salpêtrière, Université Paris VI, CNRS ESA 8149 Paris, France; 1 Laboratoire de Biochimie, Groupe Hospitalier Pitié-Salpêtrière, Université Paris VI, CNRS ESA 8149 Paris, France; 2 Biopredictive, Groupe Hospitalier Pitié-Salpêtrière, Université Paris VI, CNRS ESA 8149 Paris, France

**Keywords:** FibroScan, FibroTest, liver fibrosis, SteatoTest

## Abstract

Liver biopsy, owing to its limitations and risks, is an imperfect gold standard for assessing the severity of the most frequent chronic liver diseases chronic hepatitis C (HCV), B (HBV) non alcoholic (NAFLD) and alcoholic (ALD) fatty liver diseases. This review summarizes the advantages and the limits of the available biomarkers of liver fibrosis. Among a total of 2,237 references, a total of 14 validated serum biomarkers have been identified between 1991 and 2008. Nine were not patented and five were patented. Two alternatives to liver biopsy were the most evaluated FibroTest and Fibroscan. For FibroTest, there was a total of 38 different populations including 7,985 subjects with both FibroTest and biopsy (4,600 HCV, 1,580 HBV, 267 NAFLD, 524 ALD, and 1014 mixed). For Fibroscan, there was a total of 11 published studies including 2,260 subjects (1,466 HCV, 95 cholestatic liver disease, and 699 mixed). For FibroTest, the mean diagnostic value for the diagnosis of advanced fibrosis assessed using standardized area under the ROC curves was 0.84 (95% confidence interval 0.83-0.86), without a significant difference between the causes of liver disease, hepatitis C, hepatitis B, and alcoholic or non alcoholic fatty liver disease. High-risk profiles of false negative/false positive of FibroTest, mainly Gilbert syndrome, hemolysis and acute inflammation, are present in 3% of the populations. In case of discordance between biopsy and FibroTest, half of the failures can be due to biopsy; the prognostic value of FibroTest is at least similar to that of biopsy in HCV, HBV and ALD.

In conclusion this overview of evidence-based data suggests that biomarkers could be used as an alternative to liver biopsy for the first line assessment of fibrosis stage in the four most common chronic liver diseases, namely HCV, HBV, NAFLD and ALD. Neither biomarkers nor biopsy alone is sufficient for taking a definite decision in a given patient; all the clinical and biological data must be taken into account. There is no evidence based data justifying biopsy as a first line estimate of liver fibrosis. Health authorities in some countries have already approved validated biomarkers as the first line procedure for the staging of liver fibrosis.

The consensus in many conference statements recommend liver biopsy in the management of almost all patients with chronic liver diseases related to hepatitis C, hepatitis B, alcoholic and non alcoholic fatty liver disease. The statements have also underlined the necessity for developing reliable noninvasive tests.[1] Numerous studies strongly suggest that due to the limitations[2–8] and risks of biopsy,[9] as well as the improvement of the diagnostic accuracy of new noninvasive biomarkers, liver biopsy should no longer be considered mandatory as a first line estimate of fibrosis in the most frequent chronic liver diseases.[10,11]

Practices are evolving rapidly and a nationwide survey conducted recently in France revealed that among 546 hepatologists, 81% used noninvasive biomarker FibroTest™-ActiTest™ (FT-AT) and 32% used elastography Fibroscan™ (FS), with a dramatic decrease in the use of liver biopsy for more than 50% of the patients with chronic hepatitis C, and with a subsequent increase in the number of patients treated.[12] Furthermore, a recent overview by the French health authorities officially approved noninvasive biomarkers FT and FS as the first line estimates of fibrosis in patients with chronic hepatitis C, recommended reimbursement by social security and approved liver biopsy only as the second line estimate, in case of discordance or non interpretability of noninvasive markers.[13]

The aim of this review is to summarize the advantages and the limits of the available noninvasive biomarkers of liver fibrosis, in comparison with biopsy among patients with the four most frequently occurring chronic liver diseases - hepatitis C, hepatitis B, alcoholic and non alcoholic fatty liver disease.

## METHODS

It has been clearly demonstrated that liver biopsy, even of 40 mm length, is not a perfect gold standard; the only perfect gold standard is the entire liver or a least a sample greater than 100 mm.[4] Therefore the discordances between biopsy and a noninvasive biomarker can be due to a failure of biopsy or a failure of the biomarker.

We analyzed the published based-evidence, including the analyses of discordances and the prognostic studies.

### Design of the overview

We updated previous overviews and meta-analyses of biomarkers of advanced liver fibrosis. For serum biomarkers, the same methods were used and detailed elsewhere.[14–20] For Fibroscan, we updated the last overviews recently published.[21–22] The main non-patented and patented biomarkers have been reviewed, but the specific aim of the present overview was to focus on the professional patented fibrosis biomarkers and on Fibroscan, the most investigated transient elastography in liver diseases so far. Several previous overviews and direct comparisons have demonstrated that its panels were superior to any single biomarker, for the diagnosis of advanced fibrosis.[13–19,23–24] Several direct comparisons (including independent studies of the FT inventor) between FT, the most used patented panel and simple noninvasive tests have been published. They have all observed a greater accuracy of FT, as against Aspartate aminotransferase/Platelets Ratio Index (APRI), and of FT as viewed against Forns index.[13,26–28]

### Search strategy

We searched MEDLINE with the key words ‘liver fibrosis serum marker’ with the limit ‘human’ (July 2008). We hand-searched key journals (***Gastroenterology, Hepatology, Journal of Hepatology, Gut, Journal of Viral hepatitis*** and ***American Journal of Gastroenterology***) from February 2001 to July 2008 to validate the search, as well as the abstract books of the American Association and European Association for the Study of Liver Disease annual meetings.

### Inclusion and exclusion criteria

To select published studies, we used the Standards for Reporting of Diagnostic Accuracy (STARD) criteria and the Cochrane Database of Systematic Reviews (CDSR) methods.[29] Only publications with at least two original studies for the diagnosis of advanced fibrosis were preincluded and only patented biomarkers were analyzed in detail.

We excluded all studies except those that:

- included patients with chronic liver diseases
- stated that all patients had had the biomarker and liver biopsy
- provided data for true positives and negatives, false positives and negatives and area under the receiver operating characteristics curve (AUROC) for advanced fibrosis
- stated that the biomarker had been assessed blind to the biopsy
- stated the method used for defining the degree of fibrosis.

We were careful to avoid including data from duplicate publications. Studies published only with an abstract were excluded. We excluded biomarkers combining other nonbiochemical components such as alcohol consumption, but not the age and gender adjustments.

### Data extraction

To allow comparisons between the causes of liver disease in the studies, we categorized them into five groups: patients with CHC, CHB, ALD, NAFLD and mixed causes.

From the published study, we checked to see whether the study was performed by the biomarker inventor group (yes, no, mixed groups including inventor). Study inclusion was never dependent on the result of the noninvasive test under investigation.

### Statistical analysis

#### Diagnostic value:

The biomarker value for the diagnosis of advanced fibrosis (bridging fibrosis or stages F2, F3, F4 according to the METAVIR scoring system[30]) was assessed by the AUROC.

A significance level of 5% was used as the alpha risk. Each estimate was given with 95% confidence interval. Comparisons of the odds ratio and of percentages between strata were performed using the 95% confidence interval (95% CI). We used a random effects model for the primary meta-analysis, to obtain a summary estimate for the AUROCs with a 95% CI of biomarker, as compared with liver biopsy.

The AUROC was used as a measure of discrimination, estimated using the empirical (nonparametric) method advocated by DeLong ***et al***.[31] It was compared using the paired method recommended by Zhou ***et al***.[32] All analyses were performed on NCSS software (Kaysville, Utah, USA).

Meta-analysis was performed twice, when the details were available - once according to the absolute value of the observed AUROCs (ObAUROC) and once according to the AUROCs standardized for the spectrum of fibrosis stages (AdAUROC). We previously demonstrated that the AUROCs were definitely related to the difference between the mean fibrosis stages in the advanced fibrosis and non advanced fibrosis groups (DANA); the AdAUROC is the AUROC adjusted for the difference of the observed DANA as against a standard DANA of 2.5 fibrosis METAVIR units (DANA=2.5, if there is a uniform prevalence of 0.20 in each of the five stages); all the AUROCs were adjusted to a DANA of 2.5, using the formula: AdAUROC = ObAUROC + (0.1056) (2.5-ObDANA).[33,34]

We compared the biomarkers when there were at least two independent direct comparisons in the same disease specific population. This was possible only in patients with HCV and between FT and APRI and between FT and HepaScore (HS) and FibroMeter (FM).

#### Analyses of discordances:

Several internal and independent studies made individual assessments of the discordances observed between FibroTest-ActiTest results and histology in different chronic liver diseases.

The definition of a significant discordance between FibroTest-ActiTest and biopsy results was a discordance of at least two stages or two grades in the METAVIR scoring system. On the basis of risk factors for failure and independent endpoints, discordance was classified as being attributable to biopsy or to markers.

For FibroTest-ActiTest failure, the risk factors were hemolysis, possible Gilbert's syndrome, inflammation, acute hepatitis, and cholestasis (drug induced or extrahepatic). For biopsy failure, the risk factors were biopsy size and the number of fragments and portal tracts. Independent endpoints used for assessing extensive fibrosis and cirrhosis were platelet count, prothrombin time, dysmorphic liver on ultrasound, a reduction or reversal of portal flow, gradient portal pressure >5mmHg, and grade 2 or 3 esophageal varices. Acute flare-up of hepatitis (ALT>200U/L) was considered to represent failure of biopsy in assessing activity. Serum ferritin was used as an independent marker for necroinflammatory features. Repeated assessment of liver injury by subsequent biopsy or FibroTest-ActiTest in relation to efficacy of treatments were also considered.

In patients with chronic hepatitis B, concordant cases were defined as patients with significant disease or nonsignificant disease, presumed both with biopsy and FT-AT. In case of baseline discordance, an unexpected significant progression or improvement, in comparison with treatment efficacy or nonefficacy, strongly suggested a baseline failure of either biopsy or FT-AT.

The following rules were defined to attribute the cause of failure:

- High risk of false negative: Patients treated with adefovir and with a virological response (more than 3 logs decrease) in which the estimate of liver injury worsened were defined as high risk of baseline false negative. The definition of FT-AT worsening was an increase of at least 0.20 for FT (which corresponds to one METAVIR fibrosis stage) and 0.25 for AT (which corresponds to one METAVIR grade), and of biopsy worsening as an increase of at least one stage or one grade.
- High risk of false positive: Patients treated with placebo and without a virological response (less than 3 logs decrease) and in whom the estimate of liver injury improved were defined as high risk of baseline false positive. The definition of FT-AT improvement was a decrease of at least 0.20 for FT and 0.25 for AT. The definition of biopsy improvement was a decrease of at least one stage or one grade. The ratio between the number of discordant cases attributable to a biopsy failure and to FT-AT failure in this subpopulation of patients belonging to clearly responders or non-responders could be considered as an estimate of the overall ratio in the general population of HBV patients.

#### Prognostic value:

Severe complications (digestive hemorrhage, ascites, hepatocellular carcinoma) and mortality are very strong endpoints for assessing the accuracy of biomarkers when only imperfect gold standard is available. The prognostic values of biomarkers and biopsy were assessed using AUROCs for the prediction of liver related complications and mortality related to liver complications. A secondary endpoint was to validate that the classification of patients in three-class severity according to FT values was actually associated with mortality and morbidity.

### Role of the funding source

There was no specific financial support for this overview, but two of the authors have a potential conflict of interest: Thierry Poynard is a consultant and has a capital interest in Biopredictive, the company marketing FT, and Mona Muntenau is a full time employee of Biopredictive.

Biopredictive had no role in the study design, data collection, data analysis, data interpretation, or writing of the report. The corresponding author had full access to all the data in the study and had final responsibility for the decision to submit for publication.

### Quality evaluation of fibrosis biomarkers

A specific list of 62 items has been elaborated to assess the level of quality of each biomarker.[18,19] The aim of these items was to check the level of reliability of the published evidence-based data for each biomarker, as it is performed for a drug. Five items were related to the biomarker rational. Fourteen items were related to the sample size of validation studies. Six items were related to the preanalytical and analytical recommendations. Twenty one items were related to the benefit-risk evaluation versus liver biopsy. Two items were related to the diagnostic validation versus other endpoint than biopsy. Two items were related to the independent validation of the biomarker. Four items were related to the association of the specific fibrosis biomarker with other injury specific biomarker. Eight items were related to official approvals, availability, conflict of interest and cost.

## RESULTS

### Biomarkers identified

A total of 14 validated biomarkers have been identified [Table 1] between 1991 and 2008.[18,19] Nine were not patented: Prothrombin, Gamma Glutamyl Transpeptidase, Apolipoprotein A1 (PGA) index, Age platelet (AP) index, Bonacini index, Pohl score, Forns index, Aspartate aminotransferase/Platelets Ratio index (APRI), MP3 (MMP1, PIINP) index, FIB4, and FibroIndex. Five were patented: FT, FibroSpect II (FSP), Enhanced Liver Fibrosis (ELF), FibroMeter (FM), and HepaScore (HS). Among these panels, the number of components ranged from two to seven.[18,19]

**Table 1:** Serum markers of hepatic fibrosis with at least two validations

Index (references)	Year first publication	Key leader	Components	Liver disease
Not patented				
PGA	1991	Poynard	Prothrombin, GGT, apoA1	ALD
AP	1997	Poynard	Plt, age	HCV
Bonacini	1997	Lindsay	Plt, ALT, AST,	HCV
Pohl	2001	Pohl	Plt, AST	HCV
Forns	2002	Forns	Plt, cholesterol, age	HCV
APRI	2003	Lok	Plt, AST	HCV
MP3	2004	Leroy	PIIINP, MMP1	HCV
FIB-4	2006	Sterling	Plt, AST, ALT, age	HCV/HIV
FibroIndex	2007	Koda	Plt, AST, gamma globulins	HCV
Patented				
FibroTest/FibroSure	2001	Poynard	A2M, haptoglobin, APOA1, Bili, GGT, age, gender	HCV, HBV, ALD NAFLD, HIV
FibroSpect II	2004	Oh	A2M, HA, TIMP1	HCV
ELF	2004	Rosenberg	HA, PIIINP, TIMP1	mixed
FibroMeter	2005	Cales	Plt, AST, A2M, HA Prothrombin, age, gender	mixed
HepaScore	2005	Adams	A2M, HA, GGT, age, gender	HCV

TIMP- tissue inhibitors of metalloproteinases; MMP- matrix metalloproteinases; AST- aspartate aminotransferase; ALT- alanine aminotransferase; HA- hyaluronic acid; HOMA- homeostatic model assessment; A1- apolipoprotein A1; PI- prothrombin index; FSII- FIBROSpectII; ELF- Enhanced liver fibrosis; FT- Fibrotest; FS- Fibrosure; FM- Fibrometer. References are available in the recent published meta-analyses.[17–20] PGA- Prothrombin, Gamma Glutamyl Transpeptidase, Apolipoprotein A1; AP- Age platelet; APRI- Aspartate aminotransferase/Platelets Ratio Index; MP3- MMP1, PIINP; FIB-4- Platelet, AST, ALT, age; GGT- Gamma glutamyl transpeptidase; Plt- Platelet; PIIINP- Procollagen III; A2M- Alpha2 macroglobulin; APOA- Apolipoprotein A1; ALD- Alcoholic Liver Disease; HCV- Hepatitis C; HIV- Hepatitis B; NAFLD- Non acoholic fatty liver disease

#### FibroTest (39 studies):

**Figure 1:**
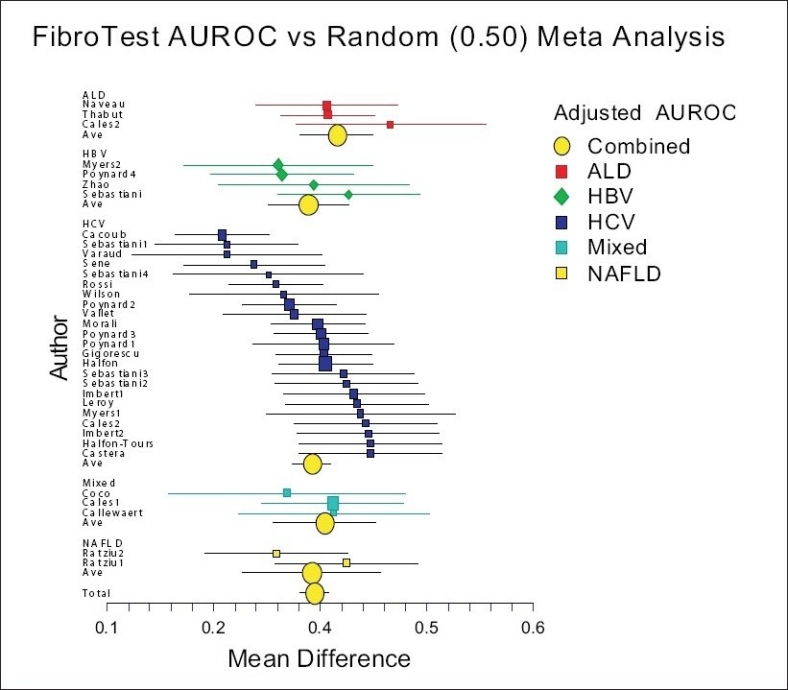
Meta-analysis of Fibrotest accuracy for the diagnosis of advanced fibrosis

For FT, a total of 39 studies (one population = one study) published in numerous articles between 2001 and 2008 were identified[17–20]; in one study, the AUROC was unknown and was not included, resulting in 38 included studies [Figure 1]. These included 7,985 subjects with both FT and biopsy (4,600 HCV, 1,580 HBV, 267 NAFLD, 524 ALD, and 1014 mixed).

#### Other serum biomarkers (12 studies):

For FSP, a total of four published studies including 463 patients were identified; two studies for ELF including 1,041 patients; three for FM including 1,134 patients and three for HS including 757 patients.[17–20]

#### Fibroscan:

For Fibroscan, a total of 15 published studies were identified[21–22] and four of them were not included for the meta-analysis concerning the diagnosis of advanced fibrosis, as the prevalence of each stage of fibrosis was not detailed or there was a suspicion of repeated publication.[35–38] These included 2,260 subjects (1,466 HCV, 95 cholestatic liver disease and 699 mixed).

### Diagnostic value and meta-analyses

#### Diagnostic value of biomarkers:

For FibroTest, the mean standardized AUROC was 0.84 (95% CI 0.82-0.86), without differences between the causes of liver diseases [Figure 1].

**Figure 2:**
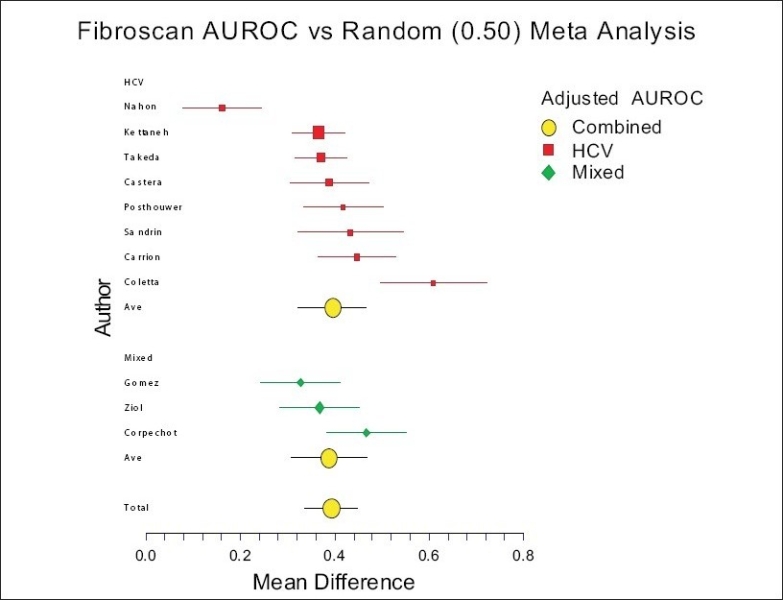
Meta-analysis of Fibrotest accuracy for the diagnosis of advanced fibrosis

For Fibroscan, the mean standardized AUROC was 0.89 (95% CI 0.84-0.95). No comparisons were possible between the causes of liver disease. There were only two or more published studies for HCV [Figure 2].

For the other patented biomarkers, the mean AUROCs were similar to those observed for FibroTest and Fibroscan.[17–20]

### Comparison between biomarkers diagnostic values

FibroTest is significantly more effective than APRI. In six studies (1,630 HCV patients), FT was directly compared with APRI,[17–20] five being independent of FT inventor. Meta-analysis demonstrated a greater observed AUROC for FT (0.83; 95% CI 0.80-0.85), as against APRI (0.76; 95% CI 0.73-0.80), with 0.06 (95% CI 0.03-0.09) mean difference (***P*** = 0.0005). This significant difference persisted when the non independent study was excluded from the analysis: 0.05 (95% CI 0.01-0.09; ***P*** = 0.003).

None of the meta-analyses of studies comparing directly patented biomarkers reached statistical significance. The number of patients was too small to detect a 5% difference between AUROCs. Between FT and HS (three studies in 653 HCV patients), the mean difference was 0.02 (95% CI - 0.03-0.07; ***P*** = 0.30) and after excluding non-independent study, it was 0.04 (95% CI - 0.01-0.08; ***P*** = 0.12). Between FT and FM (three studies in 653 HCV patients), the mean difference was -0.005 (95% CI - 0.05-0.04; ***P*** = 0.81) and after excluding non-independent study, it was 0.0008 (95% CI - 0.05-0.05; ***P*** = 0.97). Between FM and HS (two independent studies in 536 HCV patients), the mean difference was higher - 0.04 (95% CI - 0.01-0.08; ***P*** = 0.13).[17–20]

There was also no significant difference between FibroTest and Fibroscan both for advanced fibrosis and cirrhosis.[21–22]

### Discordance analysis

#### Patients with chronic hepatitis C:

In the study conducted by Poynard ***et al***., discordance was observed in 154 of 537 patients (29%), including 12% for fibrosis staging only; 12% for activity grading only and 24 patients (4.5%) for both.[39] The factors significantly associated with discordance were steatosis, an inflammatory profile, and smaller biopsy size. Discordance was attributable to markers’ failure in 13 patients (2.4%) and to biopsy failure in 97 (18%; P<0.001 versus FibroTest-AT), and was nonattributable in 44 patients (8.2%). The most frequently identified cause of FibroTest failure was false negatives attributable to inflammation, with an isolated increase in haptoglobin (four cases); one false-positive FibroTest result was attributable to hemolysis, and one to posttransplantation fibrosing cholestasis. The most frequent biopsy errors were false negatives for activity grading (10.1%) and false negatives for fibrosis staging (4.5%), both associated with smaller biopsy size and steatosis. False positives of fibrosis staging (3.5%) were associated with fragmented biopsies. In this study, the conclusion was that only a minor proportion (∼14%) of patients with chronic hepatitis C has an adequately sized liver biopsy. When biopsy and marker results are discordant, a reason can be identified in more than two-thirds of the cases and, in those cases, biopsy failure is >7 times more common than the diagnostic failure of markers.

In the Halfon ***et al***. study, discordance between FibroTest and biopsy results for fibrosis staging was observed in 92 of the 504 patients (18.4%). It was explained in half of them.[40] Discordances were attributable to FibroTest in 27 cases (5.4%) and to biopsy in 19 cases (3.8%); 46 cases (9.1%) had unexplained discrepancy. According to the risk factors, the causes of failure were considered highly attributable to FibroTest in eight cases, moderately attributable to FibroTest in 19, highly attributable to biopsy in nine, and moderately attributable to biopsy in 10.

Another study strongly suggested a higher sensitivity of the FibroTest-ActiTest, as compared to biopsy, through the analyses of the blood donor control subjects and of patients cured with antiviral treatment. In HCV infected patients who had A0 or F0 on biopsy, the values of FibroTest and ActiTest were higher than those seen in blood donors. Additionally, when these patients were cured with antiviral treatment, their FibroTest and ActiTest values decreased to the same level as that of the control patients.[41]

#### Patients with chronic hepatitis B:

In a study conducted by Poynard ***et al***., the design of the trials with an effective treatment (adefovir) and a placebo group gave the unique opportunity to identify a high-risk group of false positive or negative of FT-AT and biopsy.[42]

Among the 133 discordant cases (29% of 462), it was possible to estimate the high-risk profile of FT-AT and biopsy failure among 96 patients, who were either treated with adefovir with a virologic response (n = 63) or treated with placebo without virologic response (n = 33). Among these 96 patients, 29 had unexpected progression or improvement suggesting baseline failure of either biopsy or FT-AT. Among these 29 patients, the proportion of failure attributable to biopsy was 66% (19/29); ten out of the 19 were considered biopsy false positive (median length 11 mm) and nine as biopsy false negative high risk (median length 11 mm). The proportion of FT-AT failure was 34% (10/29). If this estimate of the proportion is true among the other discordant cases, the real rates of patients misclassified using FT-AT is not 29% as estimated by the classical analysis considering biopsy as a perfect gold standard, but 10% (34% of 29%).

#### Patients with NAFLD:

In a study conducted by Ratziu ***et al***., an analysis of group 1 (n = 170) observed discordance in 17 patients (10%): 5% due to FibroTest failure (9/170), 4% due to biopsy failure (6/170) and 1% undetermined cause (2/170).[43] In group 2 (n = 97), analysis showed a clinically significant (≥2 stage) discordance in 10 patients (10%): 2% due to FibroTest failure (2/97), 2% due to biopsy failure (2/97) and 6% undetermined cause (6/97). The present study on NAFLD patients identified 5% of discordances due to FibroTest failure, as against 4% due to biopsy failure. As previously described, Gilbert's syndrome and acute inflammation were the most frequent causes of FibroTest failures.

#### Patients with alcoholic liver disease (ALD):

In a study conducted by Naveau ***et al***., in 221 consecutive ALD patients, there was discordance of two or more fibrosis stages in 42 patients.[44] Failure was attributed to biopsy diagnosis in 26 cases: 14 false negatives and 12 false positives; failure was attributed to FibroTest in 13 cases: six false negatives and seven false positives. In three cases, the cause of failure was not determined because there were no details concerning the quality of the biopsy specimen and no isolated abnormal values of FibroTest components.

Among the 42 discordant cases, there were 18 patients with discordant diagnoses of cirrhosis; in six cases, the biopsy examination conclusion was cirrhosis while the FibroTest conclusion was of no cirrhosis. Three of the six cases were false negatives on FibroTest and three were false positives on biopsy; among the 12 discordant diagnoses of cirrhosis made by FibroTest and not by biopsy examination, three were possible false positives of FibroTest, eight were possible false negatives of biopsy examination (all poor quality), and one was undetermined. This study identified similar causes of discordances as in chronic hepatitis C. In twice as many cases, these were attributable to the poor quality of the liver biopsy rather than to the FibroTest.

### Prognostic analysis

#### FT prognostic value in HCV:

The primary aim of the prospective study of Ngo ***et al***.[45] was to assess the prognostic value of FT for 5-year HCV-related mortality and morbidity, compared to the prognostic value of histologic features of liver biopsy, the current standard reference [Table 2]. A secondary endpoint was to validate that the classification of HCV patients in the three-class severity according to FT values was actually associated with mortality and morbidity.

**Table 2:** Prognostic value of FibroTest, in patients with chronic hepatitis C, B and alcoholic liver disease

Liver disease	Number of patients	Mean duration of follow-up	No death and no liver complications AUROC (95% CI)	No liver related death	No death (overall survival)
Chronic hepatitis C	537	5 years	0.96 (0.75-0.79)	0.96 (0.75-0.79)	0.76 (0.81-0.85)
Chronic hepatitis B	1,074	4 years	0.89 (0.84-0.93)	0.95 (0.91-0.97)	0.94 (0.89-0.96)
Alcoholic liver disease	262	10 years	Not performed	0.79 (0.68-0.86)	0.69 (0.61-0.76)

All AUROCS were significantly greater (P<0.0001), than 0.50 (No prognostic value). (*n* = 1,873)

The study included a prospective hospital-based cohort of 537 chronic hepatitis C patients with liver biopsy and FT analyzed on the same day.

FibroTest classified 157 patients as severe fibrosis (FT score > 0.58), 137 as moderate fibrosis (0.32-0.58) and 243 as no or minimal fibrosis (FT <0.32). Baseline biopsy found 45% F2F3F4.

In patients with severe fibrosis (FT score >0.59), survival without HCV complications was 78.5% (95% CI= 71.2-85.9%; 28 complications], and survival without HCV-related death was 92.7% [95% CI = 88.0-97.3%; 9 HCV-related deaths). In patients with moderate fibrosis (FT score 0.32-0.58), survival without HCV complications was 98.8% [95% CI = 96.6-100%; one complication; ***P***<0.001] and survival without HCV-related death was 100% (no HCV deaths; ***P***<0.001). In patients with minimal fibrosis (FT score <0.32), these survivals were both 100% (no complications; ***P***<0.001 and no HCV deaths; ***P***<0.001).

FibroTest was a better predictor than liver biopsy histological staging for HCV complications with AUROC (95% CI) = 0.96 (0.93-0.97) vs 0.91 (0.85-0.94) [***P***=0.01] for liver biopsy and for HCV-related deaths with AUROCs= 0.96 (0.93-0.98) vs 0.87 (0.70-0.94) [***P*** = 0.046] for liver biopsy. The prognostic value of FT was significantly higher than fibrosis staging at liver biopsy multivariate regression analyses (***P***<0.01), taking into account treatment response and known prognostic factors (HIV coinfection, alcohol consumption).

Ngo ***et al***. concluded that FT had a 5-year prognostic value, at least similar to liver biopsy, being a true surrogate marker of hepatitis C severity, with a validation of the three classes of fibrosis previously defined minimal (green zone), moderate (orange zone) and severe (red zone).

#### FT Prognostic value in HBV:

The combination of transaminases (ALT), biopsy, Hepatitis B e Antibody (HBeAb) and viral load classically define the status of inactive carrier, in patients with CHB.

The first aim of the study of Ngo ***et al***. was to compare the 4-year prognostic value of FT and ActiTest to that of viral load and of biopsy (in a subpopulation with simultaneous biopsy). The second aim was to compare the 4-year prognostic value of combining FT, ActiTest and viral load for a better definition of the inactive HBV carrier status.

The main endpoint was the absence of liver-related complications. The adjustment factors were age, sex, Hepatitis B e Antigen (HBeAg), ethnic origin, alcohol consumption, HIV-Delta-HCV co-infections and treatment.

One thousand and seventy four patients with baseline FT, AT and viral load were included. The patients were 41 years old. Sixty nine percent of them was male, 47% African, 27% Asian, 20% Caucasian, and 15.5% coinfected. Mean follow-up was7.7 years (2.5 years prospective and 5.2 years retrospective). Manufacturers’ definitions were used: normal FT (≤ 0.27), normal AT (≤ 0.29). The prevalence of a 3-class viral load in I U/ml (low = <Log3, intermediate = Log3-Log5, high = >Log5) were 55, 28 and 17%, respectively. The accuracy [AUROC (95% CI)] of FT in 97 patients with simultaneous liver biopsies for the diagnosis of advanced fibrosis was similar to previous validations [AUROC = 0.83 (0.71-0.91)], and higher than ALT [0.60 (0.47-0.71)], viral load [0.53 (0.39-0.63)] (all ***P***<0.0001) and not different from the liver biopsy (***p***=ns).

At 4-years follow-up, 50 complications occurred (survival without complications was 93.4%), 36 deaths occurred (survival 95.0%), including 27 related to HBV (survival 96.1%). The prognostic value of FT was higher than those of viral load or ALT when compared using area under the ROC curves (all ***P***<0.0001), survival curves and multivariate Cox model. Among the 336 patients (without coinfection with HCV, Delta or HIV) with the classic definition of inactive carrier, 74 (22%) had advanced fibrosis presumed with FT, and three died or had complications at four years. A new definition of inactive carriers was proposed with an algorithm combining ‘zero’ scores for FT and AT (F0 and A0) and viral load classes. This new algorithm provided 100% negative predictive value for the prediction of liver related complications or death at four years.

In conclusion, the results showed an overall survival of patients with non-severe fibrosis at baseline, close to that of paired controls in the general population. In patients with severe fibrosis, the overall survival was 17% lower than that of the control population. The combination of FT together with the baseline viral load was the best combination for predicting the HBV survival without complications at 4-years, regardless of the treatment and other risk factors.

#### FT prognostic value in ALD:

In the study of Naveau ***et al***., the diagnostic and prognostic values of FT was assessed at 10 years.[47]

A total of 218 consecutive patients with ALD and available liver biopsy examination and biomarkers were included. Liver biopsy and FT were done at less than one month apart and FT assessed according to analytical recommendations. To be considered for inclusion, patients had to have consumed at least 50 g of alcohol per day, over the previous year. Biomarkers were compared using univariate [area under the diagnostic and prognostic (10-years) ROC curves (AUROCs)] and multivariate analysis (logistic regression and Cox). The median follow-up was 8.2 years, 78% male, 31% with cirrhosis, and 21% were abstinent during follow-up.

Eighty-five patients died, including 42 deaths related to liver complications. The diagnostic value (AUROC ± se) of FT for advanced fibrosis (F2F3F4) was 0.83±0.03 and cirrhosis 0.94±0.02. The prognostic values for liver disease related death of FT (AUROC = 0.79±0.04) did not differ from that of biopsy fibrosis staging (0.77±0.04). As for hepatitis C and B, the three classes of severity of fibrosis, according to FT score, permitted prediction of one low risk group (FT ≤ 0.31: 92% survival without complications), an intermediate risk (FT between 0.31-0.58: 87.5%) and a severe risk group (FT between 0.59-1: 62.6%). In multivariate analysis, adjusted on abstinence, the most significant biomarker was FT (***P*** = 0.002) and biopsy (***P*** = 0.05).

In conclusion, in patients with alcoholic liver disease, FT had similar prognostic value than biopsy, as observed in patients with chronic hepatitis C and B. Since biomarkers and biopsy similarly predicted survival or non liver-related death, biomarkers seemed to have the same rate of error as liver biopsy for baseline diagnosis.

### Quality items

For FT, the number of studies and the number of patients included were much higher than for all the five other tests. For the qualitative items, responses were missing or not satisfactory in 1/62 for FT, 32/62 for FSP, 36/62 for ELF, 32/62 for FM and 37/62 for HS.

## DISCUSSION

### Are the coauthors credible considering their possible conflict of interest?

During the last fifteen years, the first author has performed laparoscopy and liver biopsies, published extensively on the standardization of liver histology and using biopsy as main criterion, published on fibrosis progression, natural history, factors associated with fibrosis progression and on the impact of treatments. Due to the limitations of liver biopsy, including morbidity and mortality, the authors have worked on noninvasive biomarkers, in order to replace liver biopsy as the first line estimate of liver injury. After trying to develop nonpatented fibrosis biomarkers, we believe that diagnostic biomarkers must follow the same professional development, rather than drugs, with the highest levels of confidence and official approvals. Our nonpatented PGA index[48] has been prescribed confidentially in hundreds of patients in 17 years, in contrast with the patented FT that has been already prescribed in 300,000 patients in less than seven years (Biopredictive data on file, Castille personal communication). The first author has a capital interest in the startup company marketing FT but the patent belongs to the public organization ‘Assistance Publique Hôpitaux de Paris’.

### Is the perfect fibrosis biomarker possible?

Non expert physicians and patients are waiting for an almost perfect test, which is a biomarker with less than 10% of false positive or false negative results and more than 99% applicability. This is not possible, even with liver biopsy.[4] A 25 mm not fragmented biopsy is obtained in less than 50% of all large series[39] and the rate of false positive/negative of a 25 mm not fragmented biopsy is still around 20% for the diagnosis of advanced fibrosis, in comparison with the true gold standard, which is the whole liver.[4] Among the discordances observed between biopsy and biomarker estimates of fibrosis, the cause of failure is frequently due to biopsy failure.[39–47] Therefore, it is an illusion to wait for an almost perfect biomarker with an adjusted AUROC greater than 90% for the diagnosis of advanced fibrosis.[11] This point must be explained to patients and health authorities.

### Are fibrosis biomarkers effective in all chronic liver diseases?

Most of studies have been performed in patients infected with HCV and only FT has been investigated specifically in the four most frequent chronic liver diseases. For FT, meta-analysis demonstrated that the diagnostic value of FT was similar in the four most frequent chronic liver diseases. For the other tests and Fibroscan, the number of non HCV patients included was relatively small.

Is there a specific “gray zone” or “inaccurate zone” between the intermediate stages?

The different meta-analyses also demonstrated that the diagnostic value of FT for liver biopsy was similar between all the adjacent fibrosis stages, but without a specific ‘gray zone’ or ‘inaccurate zone’ between the intermediate stages. FibroTest, like biopsy, has lower diagnostic value to discriminate between two adjacent stages than between two extreme stages.[11,13,14,17–20]

The frequent statement ‘liver biopsy is still needed for definitive staging of intermediate stages’ is not evidence based. The entire liver is certainly the gold standard, but a liver biopsy of 15 mm (the median biopsy length in tertiary centers) has an AUROC of 0.82 between F1 and F2, being around 20% of false positives or false negatives.[4] Therefore, FT with an AUROC of 0.66 (usually described as a ‘weak’ value when using a true gold standard) between F1 and F2 has a relative AUROC as compared to the best AUROC possible of 0.66/0.82= 0.80, which is in the end acceptable for a noninvasive test.

The second error is the confusion between intermediate stages and adjacent stages. For any estimate of liver fibrosis, the diagnostic values (AUROCs) between adjacent stages need to be assessed. There are no significant differences in the diagnostic values (AUROCs) for FT[17] or for liver biopsy, as demonstrated by Bedossa ***et al***.,[4] according to intermediate stages as opposed to extreme stages, with the AUROCs for all adjacent stages being similar.

This, once again, underlines that assessing the AUROCs between all the adjacent stages remains the best way, knowing that for the ‘perfect’ biomarker, the best possible achievable AUROC is 0.82 for a 15 mm biopsy.

There are also different methodological approaches for the overview of fibrosis markers. Parkes ***et al***. arbitrarily defined an ‘inaccurate’ zone of a marker as that stage ‘when it cannot reliably attribute results for tests, as tests perform with lower sensitivities/specificities at thresholds where positive predictive value < 90%, negative predictive value >95%.’[49] There is no rationale for choosing these thresholds, but this definition could be acceptable if a true gold standard existed. This is not the case for fibrosis markers. If this definition is applied to 15 mm liver biopsies, the biopsy will be inaccurate in 40% of the cases for a diagnosis between F1 and F2.

### Is the liver biopsy still useful?

Yes, biopsy is still useful, but not as the first line estimate of liver injury in the four most frequent chronic liver diseases. Biopsy could be useful when validated noninvasive methods such as FT and FS are not applicable or discordant. In some countries like France, this strategy is already extensively used and approved by the health authorities.

Biopsy could be useful when several liver injuries are suspected in the same patients and in less frequent chronic liver diseases. Biomarkers and FS are so far less validated in cholestatic liver diseases (primary biliary cirrhosis and primary sclerosing cholangitis), in patients with liver transplantation, and in very rare diseases such as lymphoma or vascular liver disease. In hemochromatosis, several algorithms using biomarkers, genetic and imaging tests have been already validated as alternatives to liver biopsy.[50]

## CONCLUSION

This overview of evidence-based data suggests that biomarkers could be used as an alternative to liver biopsy for the assessment of fibrosis stage in the four most common chronic liver diseases, namely HCV, HBV, NAFLD and ALD.

Neither biomarkers nor biopsy alone is sufficient to take definitive decision in a given patient; all the clinical and biological data must be taken into account.

However, due to the dramatically insufficient risk-benefit ratio of biopsy (coefficient variation 40%, 0.3% severe adverse events and 3/10,000 mortality), it is surprising that many leaders and associations in the field of hepatology still recommend liver biopsy as the first line investigation for millions of people exposed to the risk of fibrosis. Based on current evidence, a wise recommendation would be a moratorium on liver biopsy as a first line procedure, while awaiting studies demonstrating biopsy cost-utility studied against that of biomarkers. Biopsy as a second line estimate of liver injury should still be indicated for intricate diseases or clinicobiological discordances.

***Author disclosure statement:*** Thierry Poynard is the inventor and has a capital interest in Biopredictive the company marketing FibroTest, ActiTest, SteatoTest, NashTest and AshTest. Mona Munteanu is a Biopredictive employee.
